# Supplementary material for: Association of Preoperative Prognostic Nutritional Index and Postoperative Acute Kidney Injury in Patients with Colorectal Cancer Surgery
Source: Nutrients. 2021 May 11;13(5):1604. doi: 10.3390/nu13051604 (PMC8170895; doi:10.3390/nu13051604)
Supplement: Supplementary file 1 [file nutrients-13-01604-s001.zip › supple.table 1.pdf]

Supplementary table 1. Characteristics of patients excluded from the study

	Final patients (N=3,543)	Excluded patients (N=193)	Total (N=3736)	p
Age; year	59.8 ± 11.2	58.9 ± 8.5	59.7 ± 11.0	0.196
Sex; male	2176 (61.4%)	115 (59.6%)	2291 (61.3%)	0.665
Height	1.6 ± 0.1	1.6 ± 0.1	1.6 ± 0.1	0.994
Weight	62.9 ± 10.5	60.5 ± 11.0	62.7 ± 10.5	0.002
BMI	23.8 ± 3.1	23.0 ± 3.5	23.8 ± 3.1	0.001
DM	523 (14.8%)	21 (10.9%)	544 (14.6%)	0.166
HTN	1174 (33.1%)	58 (30.1%)	1232 (33.0%)	0.419
CVA	71 (2.0%)	1 (0.5%)	72 (1.9%)	0.182
ASA status				0.651
ASA 1	840 (23.7%)	40 (20.7%)	880 (23.6%)	
ASA 2	2628 (74.2%)	149 (77.2%)	2777 (74.3%)	
ASA 3	75 (2.1%)	4 (2.1%)	79 (2.1%)	
Laparoscopic surgery	974 (27.5%)	49 (25.4%)	1023 (27.3%)	0.579
Colloid use	2551 (72.0%)	151 (78.2%)	2702 (72.3%)	0.071
Diuretics	18 (0.5%)	5 (2.6%)	23 (0.6%)	0.005
RBC transfusion	0.1 ± 0.4	0.3 ± 1.2	0.1 ± 0.5	0.021
Urine output; mL kg^{-1} hr^{-1}	1.9 ± 1.6	1.8 ± 1.6	1.9 ± 1.6	0.345
Lowest MBP; mmHg	71.0 ± 8.9	70.0 ± 9.4	71.0 ± 8.9	0.106
Operation time; min	171.1 ± 61.0	199.7 ± 93.5	172.5 ± 63.3	< 0.001
ICU admission	92 (2.6%)	6 (3.1%)	98 (2.6%)	0.840
Hospital days	8.1 ± 6.5	10.5 ± 7.8	8.4 ± 7.3	< 0.001
Overall mortality	39 (1.1%)	4 (2.1%)	43 (1.2%)	0.281
1-year mortality	25 (0.7%)	3 (1.6%)	28 (0.7%)	0.174

SD, standard deviation; BMI, body mass index; DM, diabetes mellitus; HTN, hypertension; CVA, cerebrovascular accident; ASA, American Society of Anesthesiologists classification; MBP, mean blood pressure; ICU, intensive care unit; RBC, red blood cell. Values are expressed as the mean (SD), median (interquartile range), or n (proportion).
