# Supplementary figures and images for: Association of Preoperative Prognostic Nutritional Index and Postoperative Acute Kidney Injury in Patients with Colorectal Cancer Surgery
Source: Nutrients. 2021 May 11;13(5):1604. doi: 10.3390/nu13051604 (PMC8170895; doi:10.3390/nu13051604)

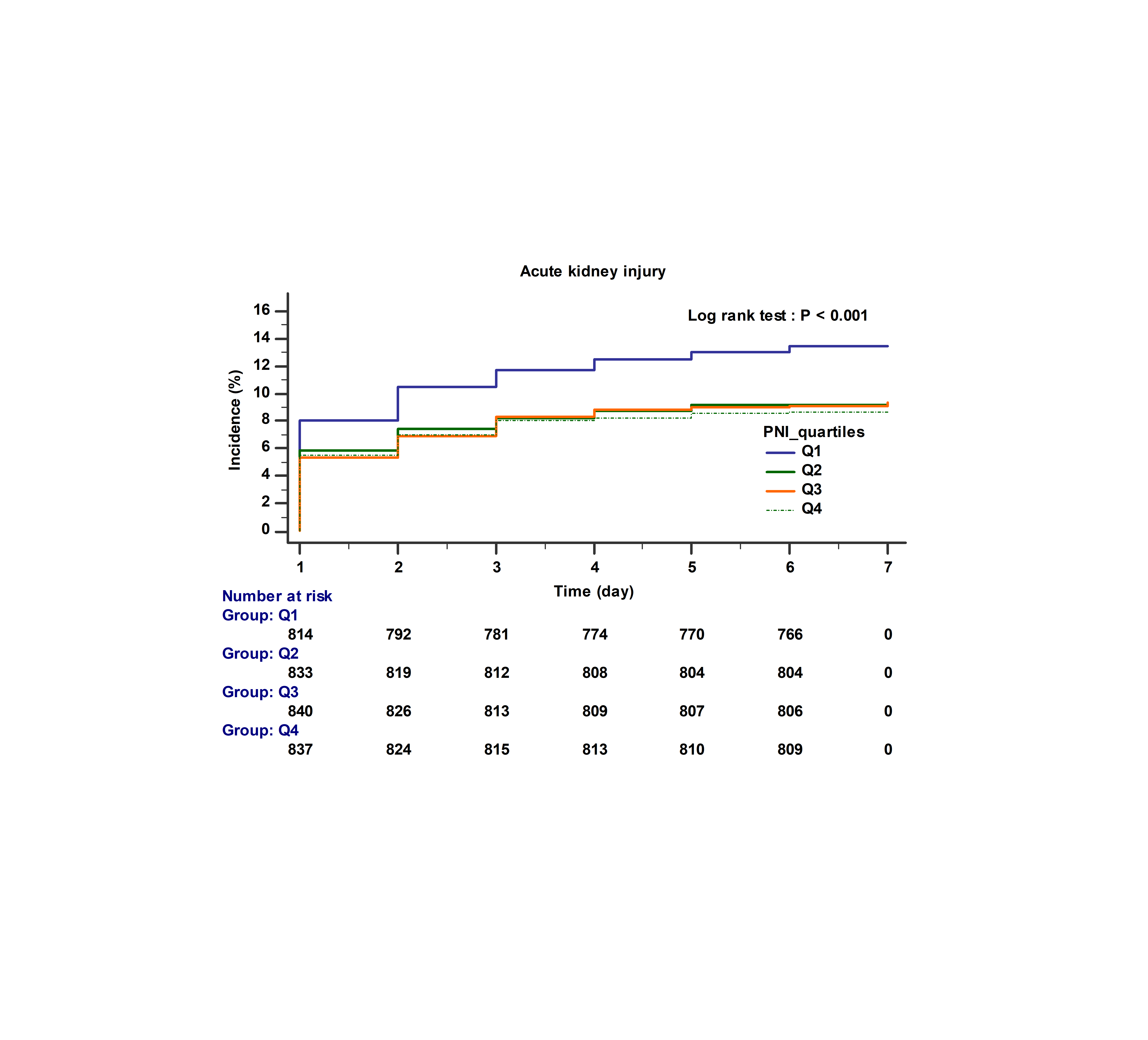

Supplement: Supplementary file 1 [file nutrients-13-01604-s001.zip › Supple.figure.1.tif]
